# Supplementary material for: Dysregulated Microglial Synaptic Engulfment in Diffuse Midline Glioma
Source: bioRxiv. 2025 Dec 25:2025.12.22.696064. Preprint. [Version 1] doi: 10.64898/2025.12.22.696064 (PMC12767537; doi:10.64898/2025.12.22.696064)
Supplement: 1 [file NIHPP2025.12.22.696064V1-supplement-1.pdf]

bioRxiv preprint doi: https://doi.org/10.64898/2025.12.22.696064; this version posted December 25, 2025. The copyright holder for this preprint (which was not certified by peer review) is the author/funder, who has granted bioRxiv a license to display the preprint in perpetuity. It is made available under aCC-BY 4.0 International license.

Supplementary Figure 1 - related to Figure 3

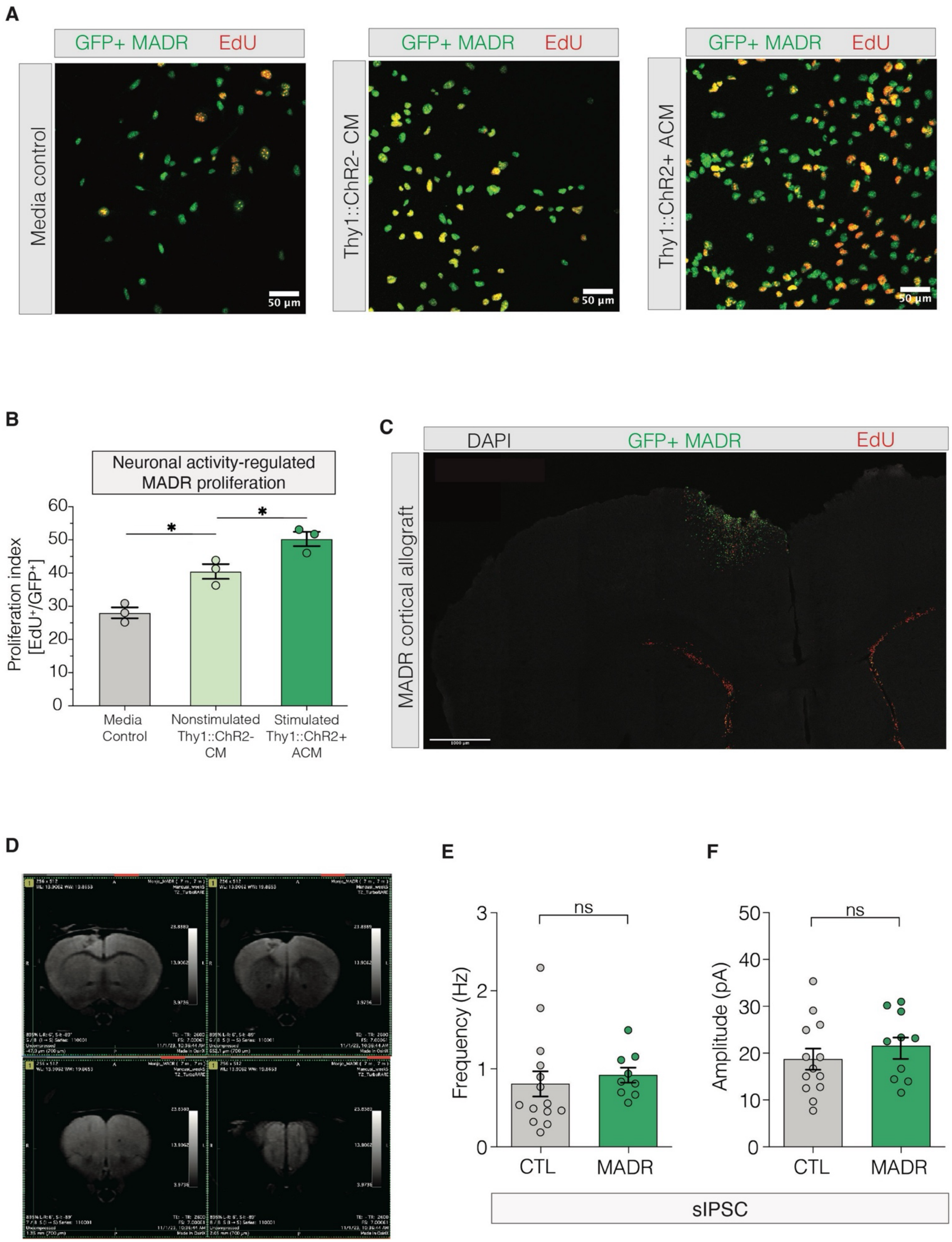

bioRxiv preprint doi: https://doi.org/10.64898/2025.12.22.696064; this version posted December 25, 2025. The copyright holder for this preprint (which was not certified by peer review) is the author/funder, who has granted bioRxiv a license to display the preprint in perpetuity. It is made available under aCC-BY 4.0 International license.

Supplementary Figure 1: Murine glioma model, MADR, conserves human glioma biological and electrophysiological features.

(A) GFP+ MADR cells (green) were incubated with aCSF (“Media control”) or ex vivo generated conditioned media from nonstimulated slices (“Thy1::ChR2-“) or from stimulated slices (“Thy1::ChR2+”). EdU (5-ethynyl-2'-deoxyuridine, red) marks dividing cells. Scale bar, 20 um.

(B) MADR cell proliferation was measured as the % of EdU+ cells of the total GFP+ MADR cells (“Proliferation Index”, y-axis), following exposure to aCSF (“Media control”), ex vivo generated conditioned media from nonstimulated slices (“Thy1::ChR2-“), or from stimulated slices (“Thy1::ChR2+”).

(C) Representative histology of cortical allografts with GFP+ MADR cells (green). DAPI (4',6-diamidino-2-phenylindole, blue) marks nuclei, EdU (red) marks dividing cells. Scale bar, 1000 um.

(D) Representative T2-enhanced MRI images (magnetic resonance imaging) of cortical MADR allografts.

(E-F) Frequency and amplitude of spontaneous inhibitory postsynaptic currents (sIPSCs) were measured.

Each individual point represents the average proliferation index of a single culture of MADR cells (B). Data are presented as mean ± s.e.m. One-way ANOVA with Tukey’s post hoc analysis (B) or unpaired t test with Welch's correction (E-F); *$P < 0.05$; NS, not significant.

bioRxiv preprint doi: https://doi.org/10.64898/2025.12.22.696064; this version posted December 25, 2025. The copyright holder for this preprint (which was not certified by peer review) is the author/funder, who has granted bioRxiv a license to display the preprint in perpetuity. It is made available under aCC-BY 4.0 International license.

Supplementary Figure 2 - related to Figure 3

A

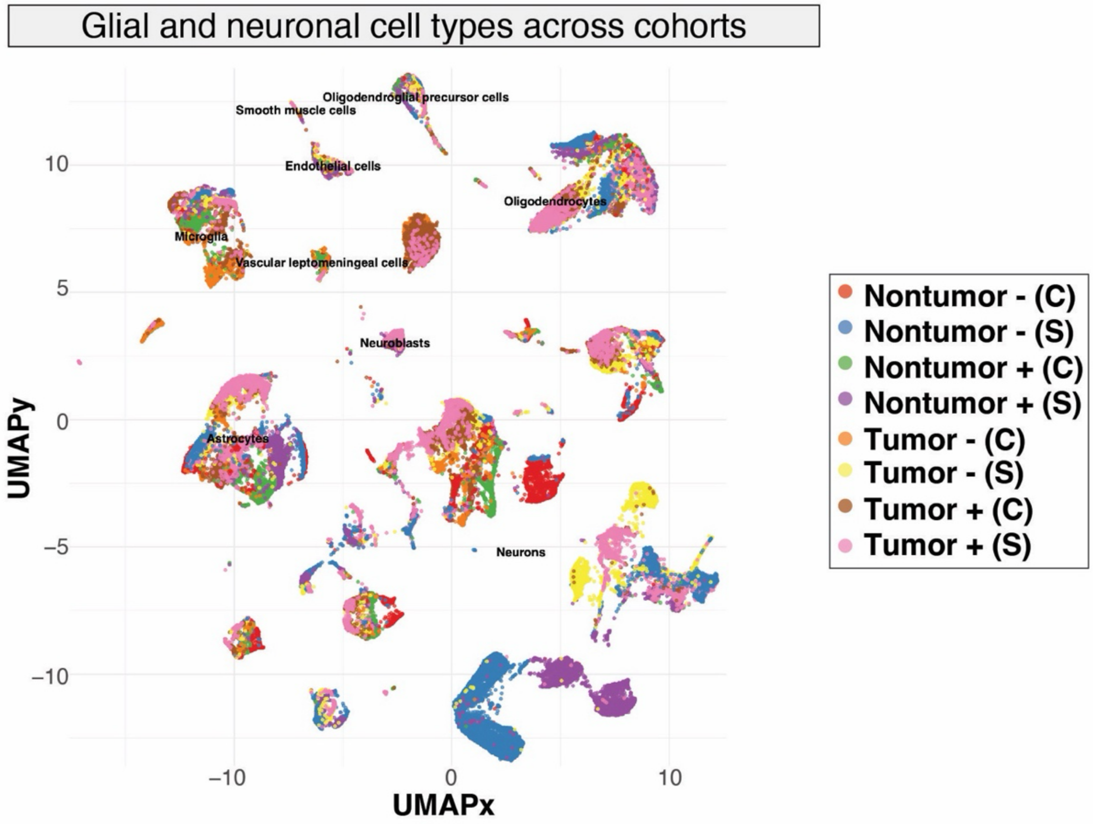

B

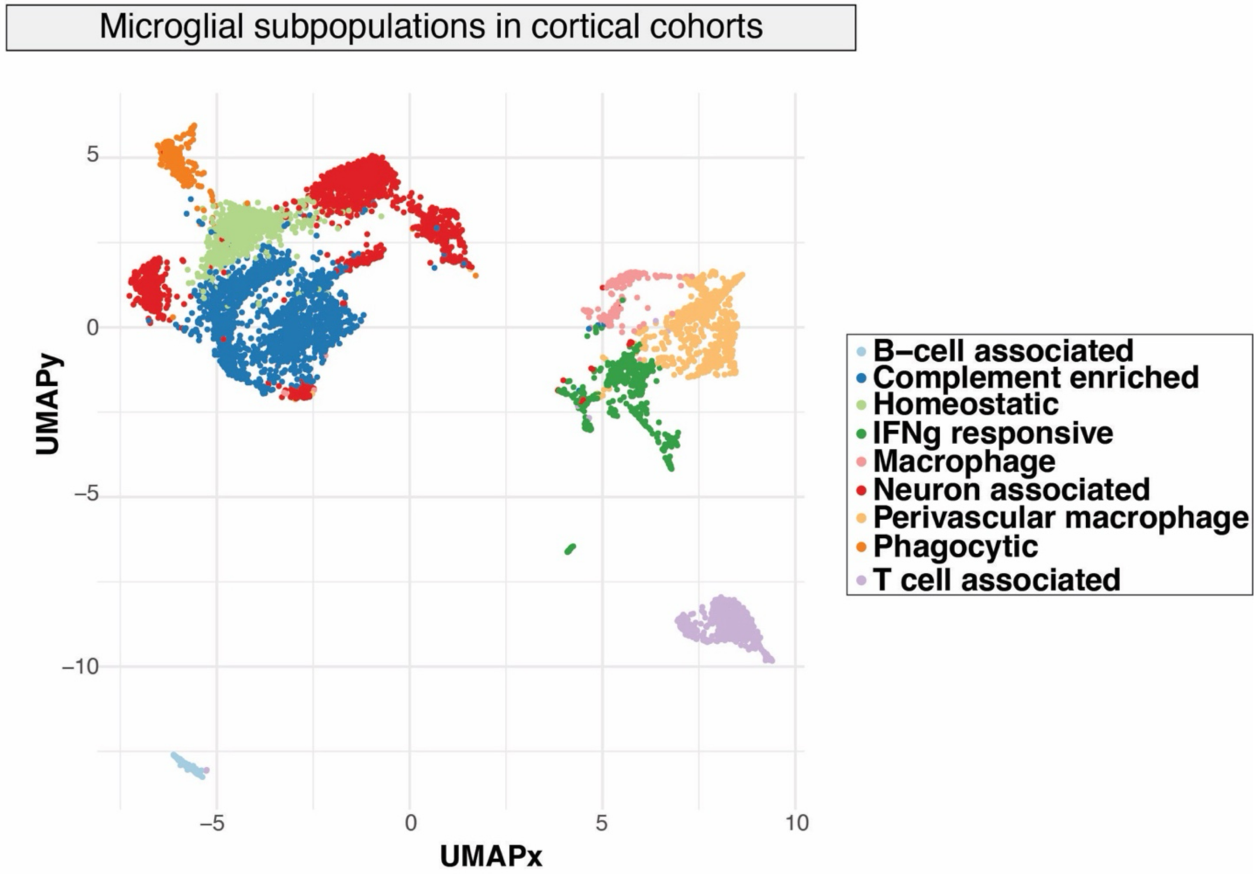

bioRxiv preprint doi: https://doi.org/10.64898/2025.12.22.696064; this version posted December 25, 2025. The copyright holder for this preprint (which was not certified by peer review) is the author/funder, who has granted bioRxiv a license to display the preprint in perpetuity. It is made available under aCC-BY 4.0 International license.

Supplementary Figure 2: Multiple cellular subpopulations identified in single nuclei sequencing dataset.

(A) Single nuclei sequencing was performed on tissue obtained from glioma allografted (“Tumor”) or saline-injected (“Nontumor”) control mice, which received cortical optogenetic stimulation (Thy1::ChR2+, “+”) or nonstimulated blue light control (Thy1::ChR2-, “-“). Both cortical (“C”) and striatal (“S”) tissue was collected from all cohorts of animals. Following Seurat-based computational analysis, single cells were mapped in a dimensionality-reduced context in 2 reduced dimensions, “UMAPy” (y-axis) and “UMAPx” (x-axis).

(B) Cells belonging to the microglial cellular cluster comprise multiple subpopulations of microglial states, with single microglial cells mapped in 2 reduced dimensions, “UMAPy” (y-axis) and “UMAPx” (x-axis).

Each point represents a single cell captured in the dataset, clustering in unique cellular populations (labeled on plot, A-B).

bioRxiv preprint doi: https://doi.org/10.64898/2025.12.22.696064; this version posted December 25, 2025. The copyright holder for this preprint (which was not certified by peer review) is the author/funder, who has granted bioRxiv a license to display the preprint in perpetuity. It is made available under aCC-BY 4.0 International license.

Supplementary Figure 3 - related to Figure 4

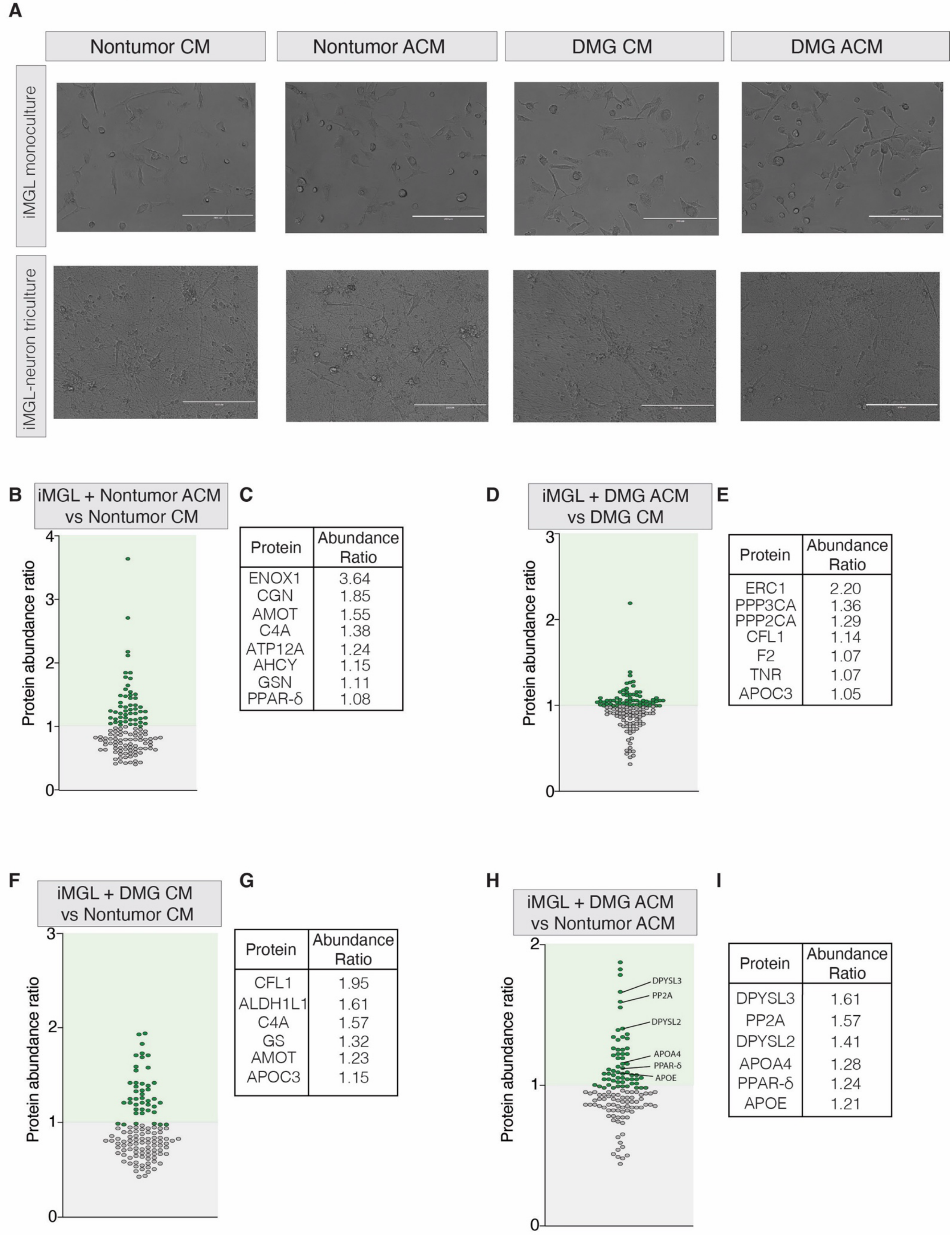

C

Protein	Abundance Ratio
ENOX1	3.64
CGN	1.85
AMOT	1.55
C4A	1.38
ATP12A	1.24
AHCY	1.15
GSN	1.11
PPAR-δ	1.08

E

Protein	Abundance Ratio
ERC1	2.20
PPP3CA	1.36
PPP2CA	1.29
CFL1	1.14
F2	1.07
TNR	1.07
APOC3	1.05

G

Protein	Abundance Ratio
CFL1	1.95
ALDH1L1	1.61
C4A	1.57
GS	1.32
AMOT	1.23
APOC3	1.15

I

Protein	Abundance Ratio
DPYSL3	1.61
PP2A	1.57
DPYSL2	1.41
APOA4	1.28
PPAR-δ	1.24
APOE	1.21

bioRxiv preprint doi: https://doi.org/10.64898/2025.12.22.696064; this version posted December 25, 2025. The copyright holder for this preprint (which was not certified by peer review) is the author/funder, who has granted bioRxiv a license to display the preprint in perpetuity. It is made available under aCC-BY 4.0 International license.

Supplementary Figure 3: Human microglia respond to glioma-secreted factors.

(A) Human iPSC-derived microglia ("iMGL") in both monoculture and neuron tri-culture change morphology in response to acute exposure to ex vivo generated conditioned media from nontumor, nonstimulated ("Nontumor CM"), nontumor, stimulated ("Nontumor ACM"), glioma-bearing nonstimulated ("DMG CM"), or glioma-bearing stimulated ("DMG ACM") slices. Scale bar, 200 um.

(B-I) Supernatant from iMGL monocultures underwent LC/MS analysis following acute exposure to the aforementioned ex vivo conditioned media, calculating the ratio of protein abundance ("Protein Abundance Ratio", y-axis) normalized on total peptide amount. Ratios are calculated by comparing iMGL supernatant after exposure to nontumor ACM and nontumor CM (B-C), glioma ACM and glioma CM (D-E), glioma CM and nontumor CM (F-G), and glioma ACM and nontumor ACM (H-I).

bioRxiv preprint doi: https://doi.org/10.64898/2025.12.22.696064; this version posted December 25, 2025. The copyright holder for this preprint (which was not certified by peer review) is the author/funder, who has granted bioRxiv a license to display the preprint in perpetuity. It is made available under aCC-BY 4.0 International license.

Supplementary Figure 4 - related to Figure 4

A

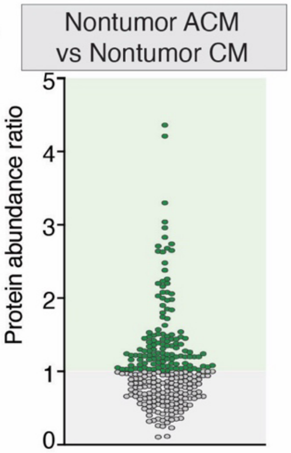

B

Protein	Abundance Ratio
VDBP	4.36
PICALM	4.21
NDPK-A	2.83
GRX3	2.38
CLIC1	2.08
CD13	2.03
NLGN3	1.98
APOE	1.53
IL-27	1.44

C

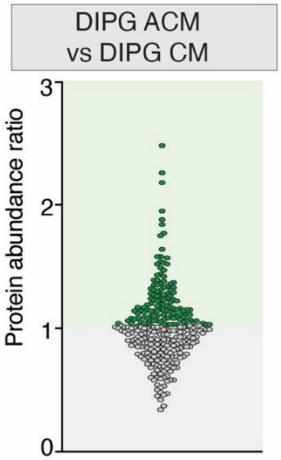

D

Protein	Abundance Ratio
PEBP1	2.48
CaMKIIβ	1.95
MBP	1.88
PFN1	1.88
MPP	1.75
CaMKIIa	1.58
PP2A	1.29
CORO1A	1.27

E

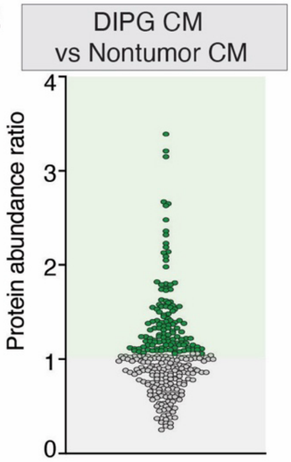

F

Protein	Abundance Ratio
NLGN3	2.05
CTL3	1.98
PPFIBP1	1.82
SERPINA3	1.55
C1Q	1.51
C2	1.38

G

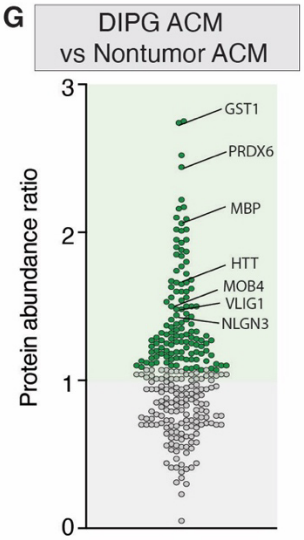

H

Protein	Abundance Ratio
GST1	2.75
PRDX6	2.52
MBP	2.16
HTT	1.62
MOB4	1.39
VLIG1	1.38
NLGN3	1.32

bioRxiv preprint doi: https://doi.org/10.64898/2025.12.22.696064; this version posted December 25, 2025. The copyright holder for this preprint (which was not certified by peer review) is the author/funder, who has granted bioRxiv a license to display the preprint in perpetuity. It is made available under aCC-BY 4.0 International license.

Supplementary Figure 4: Comprehensive analysis of ex vivo slice generated secretome.

(A-H) Ex vivo slice generated conditioned media underwent LC/MS analysis, calculating the ratio of protein abundance (“Protein Abundance Ratio”, y-axis) normalized on total peptide amount. Ratios are calculated by comparing ex vivo nontumor ACM and nontumor CM (A-B), glioma ACM and glioma CM (C-D), glioma CM and nontumor CM (E-F), and glioma ACM and nontumor ACM (G-H).

bioRxiv preprint doi: https://doi.org/10.64898/2025.12.22.696064; this version posted December 25, 2025. The copyright holder for this preprint (which was not certified by peer review) is the author/funder, who has granted bioRxiv a license to display the preprint in perpetuity. It is made available under aCC-BY 4.0 International license.

Supplementary Table 1: Microglial subpopulation gene enrichment list.

The top 50 most significantly-upregulated ($p<0.01$) genes associated with each microglial signature (B-cell associated, complement-enriched, homeostatic, IFNg responsive, macrophage, neuron associated, perivascular macrophage, phagocytic, and T cell associated, as in Fig. 3) is listed.

Supplementary Video 1: Microglial engulfment of postsynaptic structures in cortical xenograft model of pediatric high-grade glioma.

HNA+ glioma cells (SU-DIPG-VI, violet), Iba1+ microglia (white), and mCherry+ gephyrin+ synapses (red) in xenografted deep-layer premotor cortex of Thy1::ChR2 negative (“ChR2-“) mice. Scale bar, 10 um.

Supplementary Video 2: Human microglia engulf neuron-neuron postsynaptic structures in tri-culture with human GABAergic and glutamatergic neurons, following exposure to DMG-secreted factors.

Microglia (white), neurons (blue), PSD95+ excitatory synapses (red), and gephyrin+ inhibitory synapses (green) following treatment with conditioned media generated from stimulated glioma-bearing slices (“DMG ACM”). Scale bar, 20 um.
